# Viral Hepatitis and Rapid Diagnostic Test Based Screening for HBsAg in HIV-infected Patients in Rural Tanzania

**DOI:** 10.1371/journal.pone.0058468

**Published:** 2013-03-01

**Authors:** Fabian C. Franzeck, Ramadhani Ngwale, Bernadeta Msongole, Marian Hamisi, Omary Abdul, Lars Henning, Emilio Letang, Geoffrey Mwaigomole, Manuel Battegay, Christoph Hatz, Marcel Tanner

**Affiliations:** 1 Swiss Tropical and Public Health Institute, Basel, Switzerland; 2 Ifakara Health Institute, Ifakara, Tanzania; 3 University of Basel, Basel, Switzerland; 4 Division of Infectious Diseases and Hospital Epidemiology, University Hospital Basel, Basel, Switzerland; 5 St. Francis Referral Hospital, Ifakara, Tanzania; 6 Barcelona Centre for International Health Research, Hospital Clínic-Universitat de Barcelona, Barcelona, Spain; Ghent University, Belgium

## Abstract

**Background:**

Co-infection with hepatitis B virus (HBV) is highly prevalent in people living with HIV in Sub-Saharan Africa. Screening for HBV surface antigen (HBsAg) before initiation of combination antiretroviral therapy (cART) is recommended. However, it is not part of diagnostic routines in HIV programs in many resource-limited countries although patients could benefit from optimized antiretroviral therapy covering both infections. Screening could be facilitated by rapid diagnostic tests for HBsAg. Operating experience with these point of care devices in HIV-positive patients in Sub-Saharan Africa is largely lacking. We determined the prevalence of HBV and Hepatitis C virus (HCV) infection as well as the diagnostic accuracy of the rapid test device Determine HBsAg in an HIV cohort in rural Tanzania.

**Methods:**

Prospectively collected blood samples from adult, HIV-1 positive and antiretroviral treatment-naïve patients in the Kilombero and Ulanga antiretroviral cohort (KIULARCO) in rural Tanzania were analyzed at the point of care with Determine HBsAg, a reference HBsAg EIA and an anti-HCV EIA.

**Results:**

Samples of 272 patients were included. Median age was 38 years (interquartile range [IQR] 32–47), 169/272 (63%) subjects were females and median CD4+ count was 250 cells/µL (IQR 97–439). HBsAg was detected in 25/272 (9.2%, 95% confidence interval [CI] 6.2–13.0%) subjects. Of these, 7/25 (28%) were positive for HBeAg. Sensitivity of Determine HBsAg was rated at 96% (95% CI 82.8–99.6%) and specificity at 100% (95% CI, 98.9–100%). Antibodies to HCV (anti-HCV) were found in 10/272 (3.7%, 95% CI 2.0–6.4%) of patients.

**Conclusion:**

This study reports a high prevalence of HBV in HIV-positive patients in a rural Tanzanian setting. The rapid diagnostic test Determine HBsAg is an accurate assay for screening for HBsAg in HIV-1 infected patients at the point of care and may further help to guide cART in Sub-Saharan Africa.

## Introduction

Combination antiretroviral therapy (cART) has drastically reduced morbidity and mortality of HIV/AIDS in industrialized countries and recently also in resource-limited settings, including Sub-Saharan Africa (SSA) [1], [2]. Still, certain co-infections and -morbidities such as liver disease due to viral hepatitis B and C infection cause considerable morbidity and mortality: Data from the D:A:D cohort demonstrated liver-related complications to be the leading cause of non-AIDS related deaths of patients under cART in Europe, the USA and Australia in 2006 [3]. In the MACS cohort in the USA, the risk of liver-associated death was almost 10-fold increased in HIV-positive patients who had detectable hepatitis B surface antigen (HBsAg) [4].

Epidemiological data collected in rural and urban areas in various parts of Africa strongly indicate that rates of hepatitis B co-infection are higher in SSA than in Western Europe or the USA [5], [6], [7]. Due to differences in disease epidemiology e.g. the age at infection with HIV and HBV, the clinical consequences of co-infection with the hepatitis B virus (HBV) in Africa are presumably distinct from those found in industrialized countries [5]. Yet to date, liver-related outcomes in co-infected patients in SSA have only been assessed to a very limited, cross-sectional extent [8]. Nevertheless, due to a better prognosis of HIV-infection after the introduction of cART programs in SSA, an increase of patients suffering from complications of chronic viral hepatitis B, such as liver cirrhosis and hepatocellular carcinoma, could result if concurrent treatment of HBV is not addressed.

Lamivudine, emtricitabine and tenofovir are nucleoside/−tide reverse transcriptase inhibitors included in standard cART regimens in Tanzania and other countries in SSA. In addition to their suppressive effects against HIV, these drugs feature excellent antiviral activity against HBV [9]. In clinical practice, appropriate use of these agents in HIV/HBV co-infected patients results in a halt or even regression of liver cirrhosis, improvement of biological markers of disease activity and a reduction of emergence of antiviral resistance of HBV [9], [10], [11]. The World Health Organization (WHO) guidelines on antiretroviral therapy of HIV [12] recommend screening for HBV by means of HBsAg testing and the inclusion of two dually-active drugs in the antiretroviral treatment combination of co-infected patients. So far, routine screening for HBsAg is not included in the majority of national HIV programs in SSA. One reason for this might be that standard testing strategies rely on enzyme immuno assays (EIA), which require a critical and costly amount of infrastructure and human resources not available outside of urban centers. Therefore, the implementation of a viable screening strategy to identify co-infected patients is crucial as an effective intervention, i.e. cART with dually active components is already in place.

Rapid diagnostic testing (RDT) has successfully facilitated widespread screening for HIV even in rural Africa. Several point of care (POC) products for the detection of HBsAg are currently available but their take-up has been limited so far. The practical evaluation of these RDT devices in HIV-positive patients in an African peripheral setting is key in order to study the utility of including them in diagnostic routines. In this study, we prospectively assessed the diagnostic accuracy and feasibility of a scalable product locally in a large HIV outpatient clinic in rural Tanzania.

## Materials and Methods

### Patient Recruitment, Data Acquisition and Ethics Statement

Between November 2011 and June 2012, female and male, adult (≥18 years), antiretroviral-naïve, HIV-1 infected patients consecutively attending the Chronic Disease Clinic of the St. Francis Referral Hospital in Ifakara, Kilombero district, Tanzania, were prospectively offered enrollment into this study. Care at the facility was provided according to the guidelines of the national AIDS control program (NACP) [13]. Childhood vaccination for hepatitis B in this region has been introduced after the year 2002.

Patient data was retrieved from the electronic patient database of the Kilombero and Ulanga antiretroviral cohort (KIULARCO) [14]. All subjects included were given oral and written information and signed an informed consent form. Ethical approval was granted by the ethical committee of the Ifakara Health Institute (IHI-IRB Clearence #21/2011) and the Medical Research coordination Committee of the National Institute of Medical Research of Tanzania (NIMR/HQ/R.8a/Vol.IX/620-Amendment 01).

### Plasma Sample Preparation

Following inclusion into the study, 8 mL of blood were withdrawn into vacutainers containing ethylenediaminetetraacetic acid (EDTA). After completion of CD4+ cell counts and the index test (Determine HBsAg) on whole blood, samples were centrifuged for 10 min at 2000×g. Alanine aminotransferase (ALT) and creatinine levels were determined, the plasma transferred into freezer vials and stored at −80°C for later serological analysis.

### Serological Assays

All samples were tested both with the index lateral flow assay Determine HBsAg (Alere Inc., Massachusetts, USA) and the reference enzyme immunoassay Murex HBsAg 3.0 (Abbott Diagnostics, Wiesbaden, Germany). Initial reactivity in the reference assay was confirmed using the Murex HBsAg confirmatory 3.0 neutralization assay. HCV antibodies were screened using Murex anti-HCV 4.0 and HBeAg/anti-HBeAg using DiaSorin ETI-EBK plus and ETI-AB-EBK plus, respectively.

All analyses have been performed on samples obtained from one blood draw per subject. For the evaluation of Determine HBsAg, one index assay was conducted using 50 µL of whole, fresh EDTA blood, followed by a repeat index test using 50 µL of stored plasma of the identical aliquot utilized for the reference assay. Both Determine assays were performed by technicians blinded to the results of the reference test and read by two independent observers (the first being FF or RN, the second being a laboratory technician). In case of discordant readings, the result recorded by the first, i.e. higher qualified observer was used for analysis. Invalid rapid assay results (i.e. non-appearance of control band) underwent repeated testing once with the same sample. The distributors indicate sensitivity and specificity of >99.5% and >99.5% for the reference Murex HBsAg and >95.5% and >99.95% for Determine HBsAg, respectively. All EIAs were performed by FF or RN in the laboratory of IHI in Ifakara according to manufacturer’s instructions. EIA results were analyzed using automated routines in Excel (Microsoft Inc., Redmond, USA). All test kits used in this study were purchased on the open market.

CD4+ cell counting was performed using a CyFlow Counter (Partec GmbH, Münster, Germany) and analysis of ALT and creatinine with Vitrous DTSC II (Ortho-Clinical Diagnostics, Inc., Raritan, NJ).

### Statistical Analysis

SAMPLES positive for HBsAg were defined by positive reactivity in the Murex reference test followed by confirmation with the Murex neutralization assay. Prevalence of HBsAg and anti-HCV was defined as count of samples positive divided by total count of samples tested. We calculated sensitivity, specificity, negative and positive predictive values out of a 2×2 cross table using standard definitions [15]. Continuous variables are expressed as median (interquartile range) and frequencies for categorical variables. P-values of comparisons of continuous variables are derived from the Mann-Whitney-U test and from the χ^2^-test for categorical variables. Confidence intervals for proportions were calculated according to Jeffrey’s method. Inter-rater agreement is reported as Cohen’s kappa coefficient.

For the study of diagnostic accuracy, a sample size of 270 was calculated for a minimal acceptable sensitivity for Determine of 0.9, an estimated prevalence rate of 0.15, a statistical power of 0.8, and an alpha level of 0.05 [16]. To determine an estimated prevalence of disease of 15% with a precision of 0.05, a minimal sample size of 196 was ascertained. All statistical analyses were performed using Stata 11.2 (StataCorp, Texas, USA). This study has been designed and published according to the requirements of the STARD statement for reporting of studies of diagnostic accuracy [17].

## Results

### Patients Characteristics

**Figure 1:**
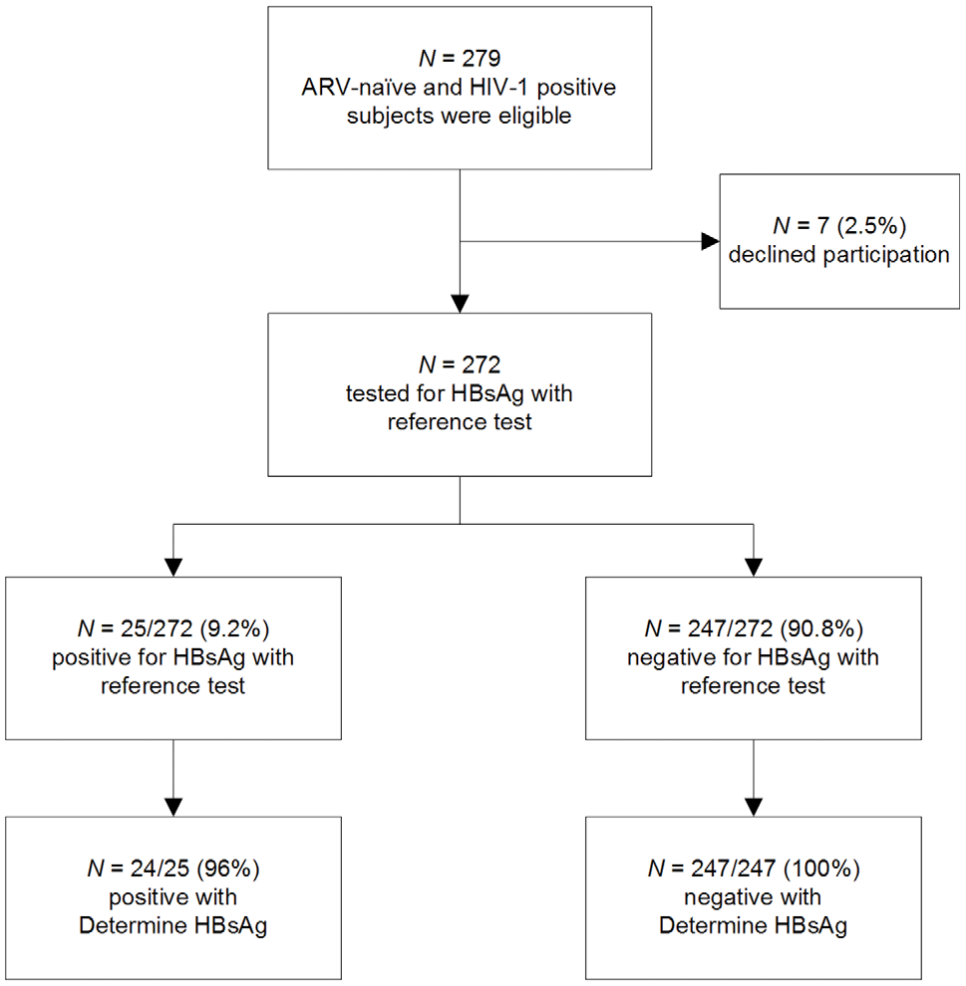
Enrollment and outcome. Study profile of the evaluation of Determine HBsAg against a reference HBsAg enzyme immuno assay.

During 8 months of enrollment, 279 adult eligible patients were offered to participate in the study as shown in the study profile in Figure 1. Characteristics of 272/279 (97.5%) consenting patients are presented in Table 1. The median age at enrollment was 38 years (interquartile range, IQR 32–47) and 169/272 (63%) of subjects were females. Patients with detectable HBsAg were significantly more likely to be male and had an elevated distribution of ALT levels (median 42 U/l, IQR 30–75 vs. 32 U/l, IQR 23–45; p = 0.006) than patients without HBsAg.

**Table 1:** Characteristics of the study population.

		HBsAg negative	HBsAg positive	Total	
		*n* = 247 (90.8%)	*n* = 25 (9.2%)	(*n* = 272)	p-value
Gender (male)		88 (35.6%)	15 (60%)	103 (37%)	0.017
Age (y)		38 (32–48)	42 (38–56)	38 (32–47)	0.18
WHO Stage	I	153 (61.9%)	12 (48%)	165 (60.6%)	
	II	51 (20.6%)	6 (24%)	57 (20.9%)	
	III	32 (12.9%)	6 (24%)	38 (13.9%)	
	IV	11 (4.4%)	1 (4%)	12 (4.4%)	0.417
CD4+ Count (/µL)		260 (99–441)	130 (76–349)	250 (97–439)	0.25
ALT (U/l)		32 (23–45)	42 (30–75)	32 (23–46)	0.006
Creatinine (µmol/L)		73 (58–86)	73 (50–93)	73 (58–86)	0.81
Anti-HCV (positive)		9 (3.6%)	1 (4%)	10 (3.7%)	0.92
HBeAg (positive)		not tested	7 (28%)	not tested	–
Anti-HBeAg (positive)		not tested	18 (72%)	not tested	–

Abbreviations: ALT, alanine aminotransferase; ARV, antiretroviral; HBe-Ag, Hepatitis B envelope antigen; HBsAg, Hepatitis B surface antigen; HCV, Hepatitis C virus; y, years.

Data are presented as median (interquartile range) for continuous variables and as count (column percentage) for categorical variables. P values hypothesis testing between HBsAg positive and HBsAg negative are derived from Mann-Whitney U test for continuous and χ2-test for categorical variables.

### Seroprevalence of Viral Hepatitis in Treatment Naïve HIV-positive Patients

HBsAg was detected in 25 of 272 (9.2%, 95% confidence interval [CI] 6.2–13.0%) subjects. Of these, 7/25 (28%, 95% CI 13.5–47.2%) were positive for HBeAg and 18/25 (72%, 95% CI 52.7–86.5%) positive for anti-HBeAg. There was no significant difference in ALT levels between HBsAg+/HBeAg+ (41 U/l, IQR 25–80, n = 7) and HBsAg+/HBeAg- (44 U/l, IQR 32–75, n = 28; p = 0.71) patients. Antibodies to HCV (anti-HCV) were found in 10/272 (3.7%, 95% CI 2.0–6.4%) of patients and 1/272 (0.36%, 95% CI 0.04–1.7%) had serological evidence of both HBsAg and anti-HCV.

### Assay Performance of Determine HBsAg Rapid Test on Whole Blood and Plasma

All 272 samples were tested both with the index and the reference test using samples of the same venipuncture. Median time to reference and index analysis of the frozen plasma samples was 25 days (IQR 8–35). All samples showed a conclusive result in Determine HBsAg upon first time execution of the assay. The performance of Determine HBsAg is shown in Table 2. Sensitivity and specificity were 96% (95% CI 82.8–99.6%) and 100% (95% CI 98.9–100%) respectively, with identical results yielded from sampling fresh blood and frozen/stored plasma. Inter-rater agreement (κ = 0.97, 95% CI 0.93–1) of the two independent readings of the rapid test was excellent with 1/272 (0.4%) result line having been rated disconcordantly due to a weak intensity coloration.

**Table 2:** Test characteristics of Determine HBsAg determined in comparison with a reference test in adult and antiretroviral-naïve HIV-1 positive patients in Ifakara, Tanzania.

TP	FP	TN	FN	Sensitivity	Specificity	NPV	PPV	Inter-rater Agreement
*N*	*n*	*n*	*n*	*% (95% CI)*	*% (95% CI)*	*% (95% CI)*	*% (95% CI)*	*κ (95% CI)*
24	0	247	1	96 (82.8–99.6)	100 (98.9–100)	99.5 (98.1–99.9)	100 (90.1–100)	0.97 (0.93–1)

Abbreviations: CI, confidence interval; FN, false negative; FP, false positive; NPV, negative predictive value; PPV, positive predictive value; TN, true negative; TP, true positive. Agreement is measured between two independent readings of Determine HBsAg.

## Discussion

The main findings of this study on 272 HIV-infected patients in a rural Tanzanian setting were a high prevalence of co-infection with viral hepatitis B and that a rapid diagnostic test for HBsAg is accurate and precise for screening for HBsAg in our setting. According to patients’ characteristics, the study population was representative for HIV-infected patients presenting for care in SSA [18].

The reported prevalence of detectable HBsAg of 9.2% in our study in HIV-positive patients in Ifakara lies within the range reported from previous studies in the same area [19] and other African settings [5]. It is significantly below the 17.3% found in an HIV outpatient clinic in Dar es Salaam, the urban hub of Tanzania [20]. The same tendency is observed when comparing the prevalence of anti-HCV antibodies of 18.1% in the urban setting, which is almost five-fold higher than the one we found. This marked variance indicates a difference in the presence and/or modification of risk factors for acquisition of viral hepatitis between rural and urban settings. Differences between these two settings have been reported in SSA before [5], [21] but there is no clear trend in which setting the odds of being infected with viral hepatitis B are higher.

A positive HBsAg status was associated with moderately increased ALT levels, which is to be expected due to the hepatic inflammatory state in patients with active replication of HBV. Of the patients with detectable HBsAg, 28% showed HBeAg positivity. A positive HBeAg status is associated with increased HBV DNA levels [7], thus being a surrogate marker of high level HBV replication. During HBV infection, HBeAg follows the appearance of HBsAg. In turn, during recovery, HBeAg clearance and seroconversion to anti-HBe precede the loss of HBsAg and detection of anti-HBsAg. Therefore it is likely that almost three quarters of our patients with detectable HBsAg were transitioning from a high replicative state to a low replicative state, with lower risk of progressive liver disease and further transmission.

This is one of the first studies prospectively evaluating a rapid test for HBsAg in HIV-infected patients entirely at the point of care in an African peripheral health institution. We chose Determine HBsAg for its low waste production, undemanding storage requirements and most importantly for its preexisting supply chains, as Determine HIV rapid tests are already part of the routine HIV testing algorithm in Tanzania [22] and many other countries. Also, the assay’s procedures and interpretation are identical with the model used for the diagnosis of HIV; this has the benefit of cutting down efforts and expenses in training staff for appropriate handling of the test in case of a large-scale deployment. Studies performed in Ghana [23] and Madagascar [24] further indicated that it can be focused on such practical factors, as differences in diagnostic accuracies between different products for the detection of HBsAg are marginal. Additionally, a recent meta-analysis [25] on Determine HBsAg test characteristics in HIV-negative subjects showed excellent sensitivity and specificity of 98.2% and 99.9%, respectively.

The few previous studies evaluating this assay in HIV-infected patients did not report encouraging findings but had limitations, notably limited sample sizes: In a study in HIV-positive patients in Kumasi, Ghana, the sensitivity was found to be only 69.3% with a specificity of 100%. The majority of samples giving a false negative result using Determine HBsAg had a concentration of HBsAg below the determined rapid test’s detection limit and a substantially lower HBV DNA viral load than samples giving a true positive result. Unfortunately, that study was retrospective in nature and results could not be linked to individual characteristics of the patients of whom the samples were originally obtained. Importantly, no information on treatment status or cART regimen was available. It can be hypothesized that present treatment with the dually-active drug lamivudine partly suppressed replication of HBV in a relevant proportion of patients to the extent that the concentration of HBsAg was below Determine HBsAg’s detection limit. In order to limit the effect of this possible interaction in our study, we only included patients with no prior cART experience. This also realistically depicts the situation where screening for HBsAg would take place mainly, namely at the inclusion into care. Two other studies on Determine HBsAg in HIV patients were consecutively conducted by a research group in Malawi. In the first study published in 2008 [26], the assay’s performance was almost random with sensitivity and specificity of 56% and 69% respectively. The authors concluded that operator errors, poor documentation and assay storage conditions might have negatively affected the results. A repetition of the study was reported in 2010 [27] with a sensitivity and specificity of both 100%. Unfortunately, only a fraction of samples negative with the index assay actually underwent testing with a reference EIA assay which introduced a verification bias. For that reason, only the statement made about specificity can be used without precaution. Additionally, the rapid testing was not conducted in the point of care setting in Malawi but in a reference laboratory in Great Britain. Yet, this result alone is a strong argument for quality issues in the first of the two studies, as specificity rose from 69% to 100% using the same assay in the identical cohort. It also re-emphasizes the need to guarantee good standards in the conduct of rapid testing, as there are pitfalls even with simple devices.

A study by Hoffmann et al. [28] investigated the performance of Determine HBsAg in HIV-infected, cART-naïve adults attending primary or antenatal care in urban South Africa. The sensitivity and specificity were rated at 75% and 100%, respectively. There was no identifiable reason reported for the only moderate level of sensitivity. The authors hypothesized that interaction with HIV infection itself might have affected performance of the assay. Reviewing the current evidence, it is not clear how sensitivity of lateral flow assays for HBsAg is affected by the concurrent presence of HIV and HBV. On one hand, to assume enhanced sensitivity seems valid, as the assay directly detects a viral protein that is expressed in higher concentrations in untreated HIV-positive compared to HIV-negative patients [29]. Serologic testing based on the demonstration of antibodies against a pathogen (e.g. anti-HCV) is more likely to be affected in patients with an altered function of the immune system [30], [31]. However, it can be hypothesized that factors impairing diagnostic performance of lateral flow rapid tests are associated with HIV infection. For example, the possible increased presence of blocking antibodies to HBsAg and immune-complex formation in the context of HIV infection and associated hypergammaglobulinemia or the prozone effect at high concentrations of the target antigen could be responsible for decreased sensitivity.

Our study provides evidence that Determine HBsAg can provide testing of good quality in HIV patients in rural Africa. It improved several limitations of previous studies by prospectively including a well characterized population at a relevant point of time, at which the assay would likely be used if adopted in routine screening. Additionally, the study was planned and reported according to accepted standards for studies of diagnostic accuracy, which some previous reports failed to achieve. However, there is a relevant degree of uncertainty about the estimates due to the limited sample size, of which previous studies also suffered. It is not clear whether its findings can be interpolated to other settings as previous evaluation of Determine HBsAg in HIV patients has shown considerable heterogeneity of sensitivity between studies.

The opportunities for HBsAg rapid testing in SSA are many [32]: HIV-infected patients chronically co-infected with HBV treated in programs offering tenofovir/emtricitabine based regimens could benefit from optimized treatment covering both infections. Even though the long-term impact of this approach on clinical outcomes could not have been studied in HIV-positive subjects in SSA so far, a reduction of complications of chronic hepatitis B and of liver-related mortality has to be expected. Treatment with dually active drugs is now recommended by the WHO [12] and other major international HIV treatment associations [33], [34]. It is the standard in more resourceful countries. The potential of improving treatment is large, as the treatment facilities and pharmaceuticals are already available in many settings. Currently, the missing link between this highly prevalent co-infection and its effective treatment above all are barriers in diagnosing it.

There are some limitations to be mentioned. Due to the cross-sectional nature of the study, the prevalence of chronic HBV infection, as defined by persistence of HBsAg for >6 months could not be established. Nevertheless, a single measurement reflects the actual field conditions prior to the initiation of cART. Also, we could not fully characterize HBV infection in our population due to the lack of other virological markers. Importantly, the number of HBsAg positive subjects included was lower than anticipated in the sample size calculation, which lead to a higher than desired level of imprecision about the estimates, particularly the sensitivity of Determine HBsAg. However, we quantified and indicated this. It must be considered that we used anticoagulated venous blood for the evaluation of Determine HBsAg and thus no direct conclusion on the performance with capillary blood can be drawn. The prevalence estimate of HCV was entirely based on detection of anti-HCV and was not confirmed by HCV RNA-PCR. This approach is likely to overestimate the true prevalence of co-infection with HCV as false positive results due to cross-reactivity in anti-HCV EIA are common in African samples [35].

This study includes also strengths. Importantly, the study was conducted prospectively and entirely at the point of care at a large out-patient clinic in rural Tanzania. The latter aspect is an essential proof of concept regarding the feasibility of Determine HBsAg at a rural site in SSA.

In conclusion, the data presented in this study demonstrate that Determine HBsAg can offer a readily available and affordable opportunity for accurate screening of viral hepatitis B in HIV patients before the initiation of antiretroviral treatment in a resource-limited setting. This provides the basis for a better quality of care and treatment of cART.
